# Nuclear ribonucleoprotein RALY targets virus nucleocapsid protein and induces autophagy to restrict porcine epidemic diarrhea virus replication

**DOI:** 10.1016/j.jbc.2022.102190

**Published:** 2022-06-24

**Authors:** Wenzhen Qin, Ning Kong, Yu Zhang, Sujie Dong, Huanjie Zhai, Xueying Zhai, Xinyu Yang, Chenqian Ye, Manqing Ye, Changlong Liu, Lingxue Yu, Hao Zheng, Hai Yu, Wen Zhang, Guangzhi Tong, Daoliang Lan, Wu Tong, Tongling Shan

**Affiliations:** 1 College of Animal & Verterinary Sciences, Southwest Minzu University, Chengdu, China; 2 Shanghai Veterinary Research Institute, Chinese Academy of Agricultural Sciences, Shanghai, China; 3 Jiangsu Co-Innovation Center for the Prevention and Control of Important Animal Infectious Disease and Zoonose, Yangzhou University, Yangzhou, China; 4 Department of Preventive Dentistry, Shanghai Ninth People’s Hospital, College of Stomatology, Shanghai Jiao Tong University School of Medicine, Shanghai, China; 5 School of Medicine, Jiangsu University, Zhenjiang, China

**Keywords:** RALY, PEDV, nucleocapsid protein, degradation, autophagy, 3-MA, 3-methyladenine, BafA1, bafilomycin A1, co-IP, coimmunoprecipitation, CRC, colorectal cancer, CQ, chloroquine, GST, glutathione-*S*-transferase, HA, hemagglutinin, HEK 293T, human embryonic kidney 293T cell line, MARCH8, membrane-associated RING-CH 8, MOI, multiplicity of infection, N, nucleocapsid protein, NDP52, nuclear dot protein 52 kDa, PEDV, porcine epidemic diarrhea virus, qRT–PCR, quantitative real-time PCR

## Abstract

Porcine epidemic diarrhea virus (PEDV) causes diarrhea and dehydration in pigs and leads to great economic losses in the commercial swine industry. However, the underlying molecular mechanisms of host response to viral infection remain unclear. In the present study, we investigated a novel mechanism by which RALY, a member of the heterogeneous nuclear ribonucleoprotein family, significantly promotes the degradation of the PEDV nucleocapsid (N) protein to inhibit viral replication. Furthermore, we identified an interaction between RALY and the E3 ubiquitin ligase MARCH8 (membrane-associated RING-CH 8), as well as the cargo receptor NDP52 (nuclear dot protein 52 kDa), suggesting that RALY could suppress PEDV replication by degrading the viral N protein through a RALY–MARCH8–NDP52–autophagosome pathway. Collectively, these results suggest a preventive role of RALY against PEDV infection *via* the autophagy pathway and open up the possibility of inducing RALY *in vivo* as an effective prophylactic and preventive treatment for PEDV infection.

Porcine epidemic diarrhea virus (PEDV), a member of the genus *Alphacoronavirus* in the family Coronaviridae, causes acute diarrhea, vomiting, dehydration, and high mortality in neonatal piglets (1). PEDV has a single-stranded positive-sense RNA genome of approximately 28 kb (excluding the poly A-tail), encoding for four structural proteins (spike [S], envelope [E], membrane [M], and nucleocapsid [N] proteins), 16 nonstructural proteins (nsp1–nsp16), and an accessory protein ORF3 (2). Moreover, porcine epidemic diarrhea has been reported worldwide, causing huge economic losses to the swine industry over the past 30 years (3). Hence, investigation on new antiviral prophylactic and treatment strategies to restrict PEDV infection is necessary.

RALY (also known as hnRNPCL2) is a member of the heterogeneous nuclear ribonucleoprotein family of RNA-binding proteins that play an important role in mRNA splicing and metabolism (4). The biological functions of RALY have been widely explored. Furthermore, RALY is shown to be associated with embryonic lethality in homozygous lethal yellow mice (5, 6). The gene *RALYL* (RALY RNA-binding protein-like) was shown to increase hepatocellular carcinoma stemness by sustaining the mRNA stability of the transforming growth factor β2 (7). Moreover, RALY may be involved in the biological processes linked with tumorigenesis and promote the proliferation of breast cancer (8), non–small-cell lung cancer (9), hepatocellular carcinoma (10), and cervical cancer cells (11).

Autophagy is a self-degradative process that eliminates unnecessary or dysfunctional intracellular components and is critical for maintaining cellular homeostasis during stressful conditions, such as cellular starvation, infection, or organelle damage (12, 13). Autophagic pathways can act as an antiviral defense mechanism (13, 14, 15, 16). PEDV N protein plays an important role in counteracting the host's innate immunity to promote viral replication (17). Therefore, N protein is important in the PEDV replication and may be a potential target to inhibit virus replication by host's innate immunity. Our previous studies have demonstrated that host factors, such as EGR1 (early growth response gene 1) (18), PABPC4 (poly(A)-binding protein C4) (18), TRIM21 (tripartite motif protein 21) (19), and BST2 (bone marrow stromal cell antigen 2) (20), can suppress PEDV replication by targeting and degrading the viral N protein *via* selective autophagy. This observation led to the hypothesis that the anti-PEDV property of RALY is linked to induction of autophagy.

In the present study, we aimed to better understand the host resistance mechanism in PEDV infection by investigating the molecular mechanisms regulated by RALY upon PEDV infection. We found that RALY suppressed PEDV replication by promoting the autophagic degradation pathway. Thus, RALY may act as a potential therapeutic target in PEDV infection prevention.

## Results

### Correlation between RALY expression and PEDV-mediated infection

**Figure 1:**
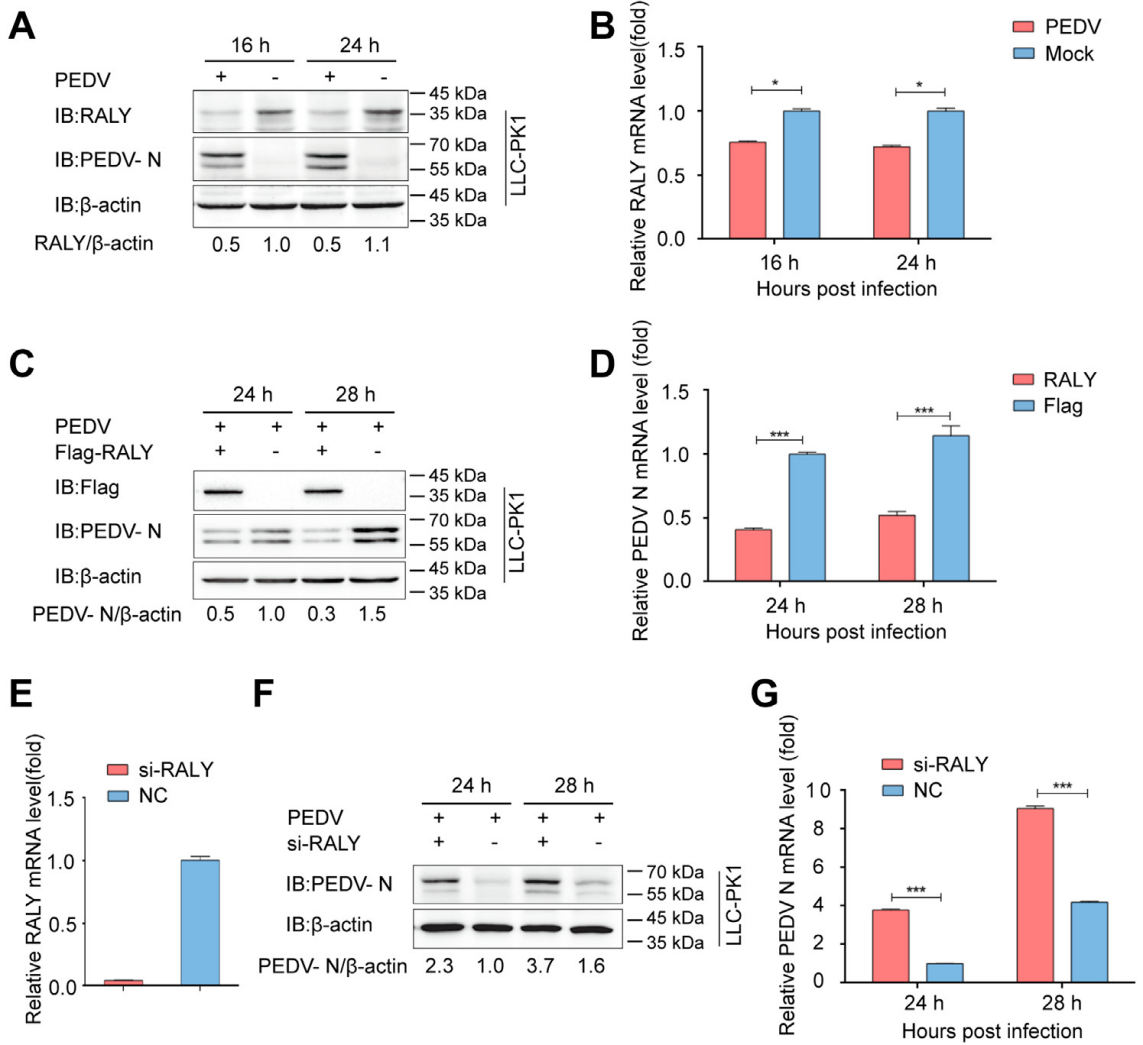
**The relationship of RALY and PEDV.***A*, LLC-PK1 cells were infected with PEDV at an MOI of 1 and analyzed at 16 and 24 hpi. The expression of RALY and PEDV N proteins was analyzed by Western blotting. β-actin was used as the sample loading control. *B*, the mRNA level of RALY was also analyzed by qRT–PCR. *C* and *D*, FLAG-RALY plasmid was transfected into LLC-PK1 cells, which were then infected with PEDV at an MOI of 1 at 24 h post-transfection. PEDV N protein was analyzed at 24 and 28 h postinfection with Western blotting and qRT–PCR. *E*, the siRALY was transfected into LLC-PK1 cells, and the knockdown efficiency of the RALY siRNA was analyzed by real-time PCR. *F* and *G*, LLC-PK1 cells were transfected with RALY siRNA or negative control siRNA. At 24 h post-transfection, cells were infected with PEDV at an MOI of 1. PEDV N protein was analyzed at 24 and 28 h postinfection with Western blotting and qRT–PCR, respectively. Data are means ± SD of triplicate samples. ∗*p* < 0.05, two-tailed Student’s *t* test. MOI, multiplicity of infection; N, nucleocapsid protein; PEDV, porcine epidemic diarrhea virus; qRT–PCR, quantitative real-time PCR.

To investigate the effects of PEDV infection on RALY expression, we analyzed the mRNA and protein levels of RALY during viral infection. Pig kidney epithelial cells, LLC-PK1, were first infected with PEDV (strain JS-2013) at a multiplicity of infection (MOI) of 1, as previously described (21). Infected cells were collected, and the expression of RALY was evaluated by Western blotting and quantitative real-time PCR (qRT–PCR) at 16 and 24 h post-PEDV infection (hpi). The results indicated that both protein and mRNA levels of RALY were significantly suppressed in PEDV-infected LLC-PK1 cells compared with those in mock-infected cells (Fig. 1, *A* and *B*). This finding suggests that PEDV infection may inhibit endogenous RALY expression in host cells.

RALY has been considered to regulate colorectal cancer (CRC) cell proliferation and apoptosis as an RNA-binding protein (22) and play an important role in mRNA splicing and metabolism (9).To study the effect of RALY in PEDV infection, LLC-PK1 cells were transfected with FLAG-RALY plasmid and, after 24 h, infected with PEDV at an MOI of 1. Western blotting and qRT–PCR analyses showed that PEDV N protein and mRNA levels were significantly downregulated at 24 and 28 hpi in LLC-PK1 cells (Fig. 1, *C* and *D*). Moreover, when LLC-PK1 cells were transfected with specific siRNAs targeting RALY (Fig. 1*E*), the replication of the virus increased upon silencing RALY (Fig. 1, *F* and *G*). These results suggest that RALY can suppress PEDV replication in LLC-PK1 cells.

### RALY suppresses PEDV replication in Vero cells

**Figure 2:**
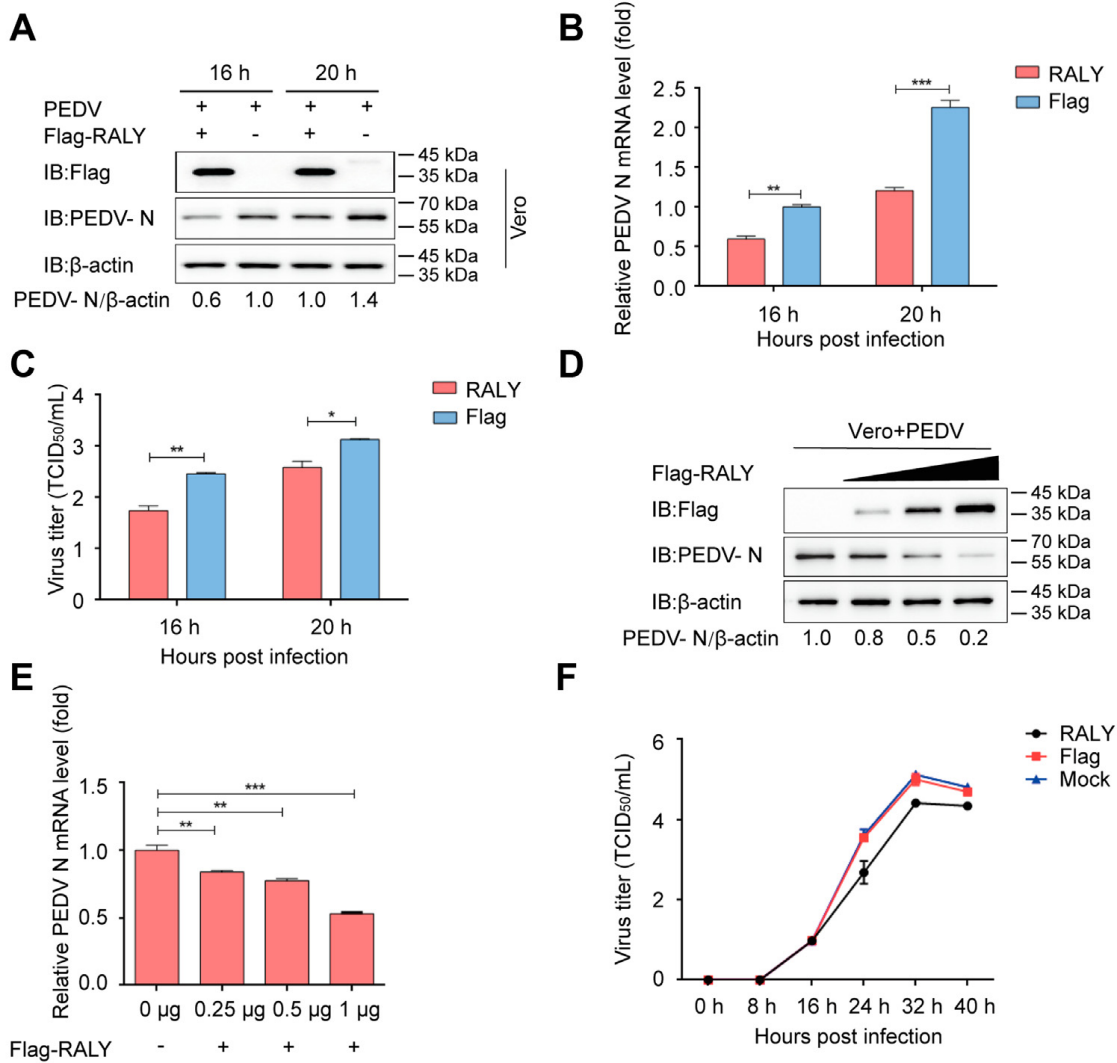
**RALY suppresses PEDV replication.***A* and *B*, RALY plasmids were transfected into Vero cells, which were then infected with PEDV at an MOI of 0.01 at 24 h post-transfection. PEDV replication was analyzed at 16 and 24 h postinfection with Western blotting and qRT–PCR, respectively. *C*, PEDV titers in the culture supernatants of the Vero cells treated described in (*A*) were measured as TCID_50_. *D* and *E*, Vero cells were transfected with increasing concentrations of FLAG-RALY plasmids and infected with PEDV at an MOI of 0.01. Cell lysates and culture supernatants were collected for analysis of PEDV replication with Western blotting and qRT–PCR, respectively. *F*, Vero cells were transfected with FLAG-RALY plasmids and infected with PEDV at an MOI of 0.01. The culture supernatants were harvested at the indicated times and detected the viral titers as TCID_50_. Data are means ± SD of triplicate samples. ∗*p* < 0.05, ∗∗*p* < 0.01, ∗∗∗*p* < 0.001, two-tailed Student’s *t* test. MOI, multiplicity of infection; PEDV, porcine epidemic diarrhea virus; qRT–PCR, quantitative real-time PCR; TCID_50_, 50% tissue culture infective dose.

To further define the inhibitory effect of RALY on PEDV replication, an independent cell line, the monkey kidney epithelial cells (Vero cells), was transfected with FLAG-RALY plasmid and, after 24 h, infected with PEDV at an MOI of 0.01. Both infected cells and their culture supernatants were collected at 16 and 20 hpi and analyzed for PEDV N expression and viral load, respectively. A reduction in viral load was observed at 16 and 20 hpi using Western blotting (Fig. 2*A*), qRT–PCR (Fig. 2*B*), and the median 50% tissue culture infective dose (Fig. 2*C*). The results demonstrated that the overexpression of RALY significantly inhibited PEDV replication. Furthermore, the inhibitory effect on viral replication appeared directly correlated with RALY expression level as the N protein level decreased with the increased expression of FLAG-RALY (Fig. 2, *D* and *E*). The viral titers in the supernatants of the infected Vero cells confirmed that the increased expression of RALY suppressed PEDV replication over time (Fig. 2*F*). Overall, our data indicate that RALY may negatively regulate PEDV replication in Vero cells.

### RALY interacting with PEDV N protein degrades N *via* autophagy

**Figure 3:**
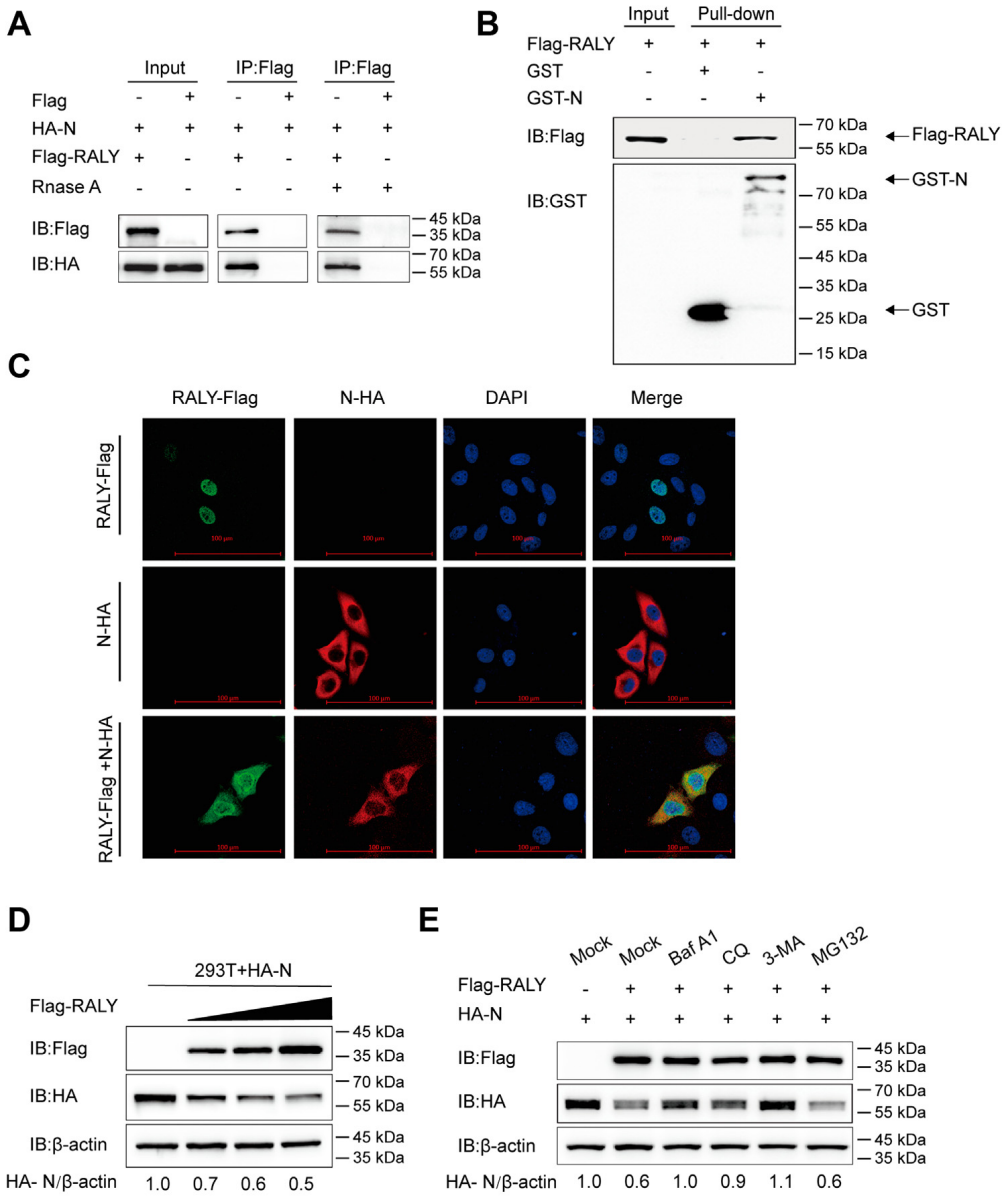
**RALY interacting with PEDV N protein degrades N by autophagy.***A*, HEK 293T cells were transfected with plasmids encoding HA-N and FLAG-RALY for 24 h, followed by co-IP with anti-FLAG binding beads. The precipitated proteins were analyzed by Western blotting analysis. The interaction of FLAG-RALY with PEDV N protein was also analyzed after RNase treatment. *B*, the RALY and PEDV N were cloned into pCold TF plasmid and pCold GST plasmid and expressed in bacterial strain BL21 (DE3) for the GST affinity–isolation assay. The eluted proteins were analyzed by Western blotting. *C*, HeLa cells were transfected with plasmids encoding FLAG-RALY and HA-N for 24 h and labeled with specific primary and secondary antibodies. Cell nuclei were labeled with 4,6-diamidino-2-phenylindole (DAPI), and the fluorescent signals were observed using confocal immunofluorescence microscopy. The scale bars represent 100 μm. *D*, HEK 293T cells were transfected with increasing concentrations of FLAG-RALY plasmid and HA-N. N protein abundance was analyzed by Western blotting. *E*, HEK 293T cells were transfected with plasmids encoding FLAG-RALY plasmid and HA-N and treated with BafA1 (50 μM), CQ (50 μM), 3-MA (1 mM), and MG132 (5 μM), respectively. Cell lysates were analyzed by Western blotting. BafA1, bafilomycin A1; co-IP, coimmunoprecipiation; CQ, chloroquine; GST, glutathione-*S*-transferase; HA, hemagglutinin; HEK 293T, human embryonic kidney 293T cell line; 3-MA, 3-methyladenine; N, nucleocapsid protein; PEDV, porcine epidemic diarrhea virus.

To explore the biological mechanism used by RALY to regulate PEDV replication, we analyzed the interaction of RALY with PEDV structural proteins (S1, S2, E, M, and N). A coimmunoprecipitation (co-IP) assay was used after cotransfecting human embryonic kidney 293T (HEK 293T) cells with plasmids encoding for each PEDV structural protein and FLAG-RALY constructs. PEDV N protein was found to be precipitated by FLAG-RALY (Fig. 3*A*), and the interaction was not affected by the inclusion of RNase in the cell lysis process (Fig. 3*A*), thus indicating that PEDV N protein interacts directly with RALY, independent from the presence of RNA. In addition, a glutathione-*S*-transferase (GST) pull-down assay was performed, which revealed binding between PEDV N (GST-N) and RALY (Fig. 3*B*). Furthermore, HeLa cells were cotransfected with FLAG-RALY and hemagglutinin (HA)-N, and protein localization was examined 24 h post-transfection. As shown by confocal microscopy, RALY was predominantly localized in the nucleus and then shuttled to the cytoplasm to efficiently colocalize with N proteins in the cytoplasm (Fig. 3*C*). In summary, these data demonstrated that RALY interacts with the N protein of PEDV.

Coronavirus N protein performs multiple functions in the viral replication cycle and pathogenesis, such as the host cell cycle regulation and immune system interference (23). To identify the mechanism by which RALY interacts with the PEDV N protein, we cotransfected HEK 293T cells with plasmids encoding FLAG-RALY and HA-N and, after examining the expression of N protein, we found that RALY suppressed N protein expression in a dose-dependent manner (Fig. 3*D*). Moreover, after FLAG-RALY/HA-N cotransfection, HEK 293 cells were treated with the protease inhibitor MG132 or the autophagy inhibitors bafilomycin A1 (BafA1), 3-methyladenine (3-MA), and chloroquine (CQ). Western blotting results showed no change after MG132 treatment, whereas the degradation of N protein was reversed by the autophagy inhibitors BafA1, 3-MA, and CQ (Fig. 3*E*). Collectively, these results demonstrated that RALY promotes the degradation of PEDV N protein through autophagy.

### RALY degrades PEDV N protein through the RALY–membrane-associated RING-CH 8–nuclear dot protein 52 kDa–autophagosome pathway

**Figure 4:**
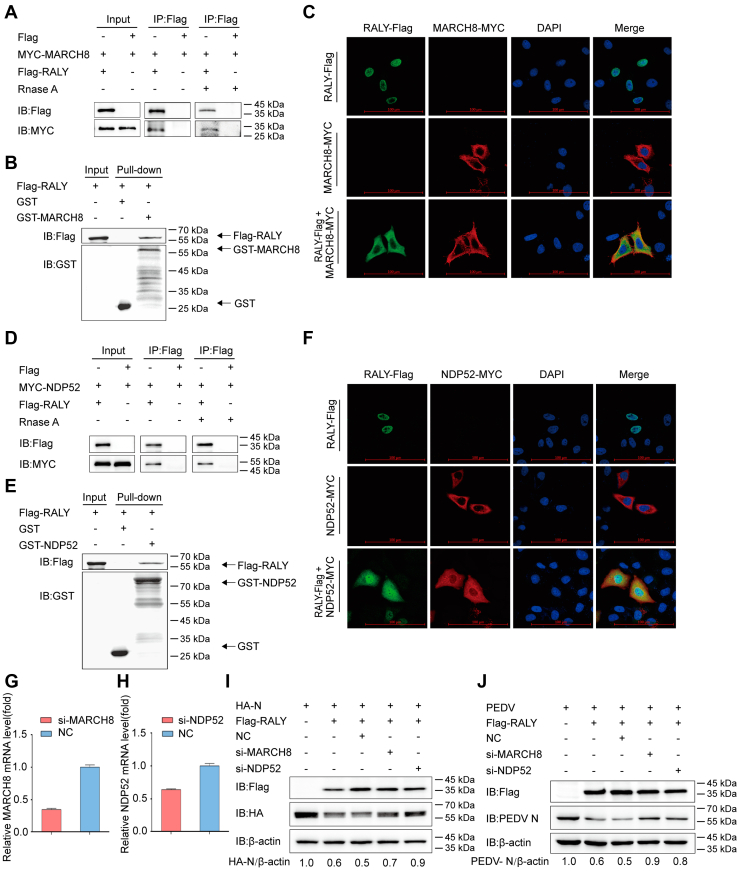
**RALY degrades PEDV N protein through the RALY–MARCH8–NDP52–autophagosome pathway.***A*, HEK 293T cells were transfected with plasmids encoding MYC-MARCH8 and FLAG-RALY for 24 h, followed by co-IP with anti-FLAG binding beads. Precipitated proteins were analyzed by Western blotting analysis. The interaction of FLAG-RALY with MARCH8 was also analyzed after RNase treatment. *B*, the RALY and MARCH8 were expressed in bacterial strain BL21 (DE3) for the GST affinity–isolation assay. The eluted proteins were analyzed by Western blotting. *C*, HeLa cells were transfected with plasmids encoding FLAG-RALY and MYC-MARCH8 and labeled with specific primary antibodies and secondary antibodies. Cell nuclei were labeled with DAPI, and the fluorescent signals were observed using confocal immunofluorescence microscopy. Cells were transfected with plasmids encoding FLAG-RALY or MYC-MARCH8 as control. The scale bars represent 100 μm. *D*, HEK 293T cells were transfected with plasmids encoding MYC-NDP52 and FLAG-RALY, followed by co-IP with anti-FLAG binding beads. Precipitated proteins were analyzed by Western blotting analysis. The interaction of FLAG-RALY with MARCH8 was also analyzed after RNase treatment. *E*, the RALY and NDP52 were expressed in bacterial strain BL21 (DE3) for the GST affinity–isolation assay. The eluted proteins were analyzed by Western blotting. *F*, HeLa cells were transfected with plasmids encoding FLAG-RALY and MYC-NDP52 and labeled with specific primary and secondary antibodies. Cell nuclei were labeled with DAPI, and the fluorescent signals were observed using confocal immunofluorescence microscopy. The scale bars represent 100 μm. *G* and *H*, the siMARCH8 or siNDP52 was transfected into HEK 293T cells, and the knockdown efficiency of the MARCH8 siRNA or NDP52 siRNA was analyzed by real-time PCR. *I*, HEK 293T cells were cotransfected with plasmids encoding HA-N, FLAG-RALY, and MARCH8 siRNA or NDP52 siRNA or negative control (NC) siRNA. N protein abundance was analyzed by Western blotting. *J*, Vero cells were transfected with plasmids encoding FLAG-RALY and MARCH8 siRNA or NDP52 siRNA or NC siRNA. At 24 h post-transfection, cells were infected with PEDV at an MOI of 0.01, and cell lysates were collected for analysis of PEDV N protein expression with Western blotting. co-IP, coimmunoprecipitation; DAPI, 4,6-diamidino-2-phenylindole; GST, glutathione-*S*-transferase; HA, hemagglutinin; HEK 293T, human embryonic kidney 293T cell line; MARCH8, membrane-associated RING-CH 8; MOI, multiplicity of infection; N, nucleocapsid protein; NDP52, nuclear dot protein 52 kDa; PEDV, porcine epidemic diarrhea virus.

In our previous study, we reported that autophagy was induced by PEDV infection (24). Moreover, several host antiviral factors, such as BST2 and PABPC4, could recruit E3 ubiquitin ligase MARCH8 (membrane-associated RING-CH 8) to mediate PEDV N protein ubiquitination, and the ubiquitinated N protein could be recognized by the cargo receptor NDP52 (nuclear dot protein 52 kDa) and delivered to the lysosome for degradation (19, 24). To investigate the mechanism of RALY-induced degradation of PEDV N *via* autophagy, we performed a co-IP assay (Fig. 4*A*) and found that FLAG-RALY coimmunoprecipitated with the E3 ubiquitin ligase MARCH8, and which is not interfered by cell lysis with RNase. The GST affinity–isolation assay and immunofluorescence confocal microscopy analysis further confirmed the direct binding of RALY to MARCH8 and their colocalization in the cytoplasm (Fig. 4, *B* and *C*).

Next, we examined the interactions between RALY and the cargo receptor NDP52. The binding of RALY with NDP52, which is not interfered by cell lysis with RNase, was observed by both co-IP assay (Fig. 4*D*) and GST affinity–isolation assays (Fig. 4*E*). The immunofluorescence confocal microscopy analysis also showed that RALY transferred from the nucleus to the cytoplasm and colocalized with NDP52 (Fig. 4*F*).

To further confirm that the MARCH8–NDP52–autophagosome pathway is necessary for RALY-induced autophagic PEDV N protein degradation, HEK 293T cells were cotransfected with HA-N and FLAG-RALY, together with either MARCH8 siRNA or NDP52 siRNA (Fig. 4, *G* and *H*). Western blotting revealed that depletion of MARCH8 or NDP52 inhibited RALY-induced degradation of PEDV N protein (Fig. 4*I*). Moreover, we cotransfected MARCH8 siRNA or NDP52 siRNA into RALY-overexpressing Vero cells and, after 24 h, infected the cells with PEDV at an MOI of 0.01. The results indicated that depletion of either MARCH8 or NDP52 restored the RALY-mediated inhibition of PEDV infection (Fig. 4*J*). These results consistently indicate that RALY degrades PEDV N protein through the RALY–MARCH8–NDP52–autophagosome pathway.

## Discussion

**Figure 5:**
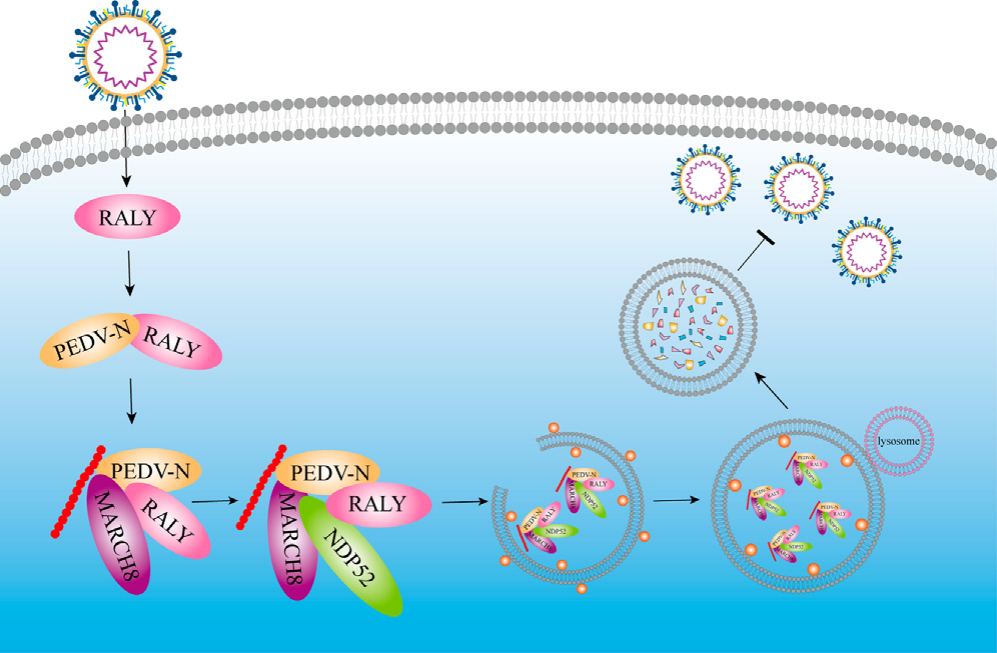
**Antiviral mechanism of RALY inhibiting PEDV replication.** During PEDV infection, RALY interacts with E3 ubiquitin ligase MARCH8 to ubiquitin viral N protein and recruits the cargo receptor NDP52 to recognize the ubiquitinated N protein and deliver it to autophagosome for degradation. MARCH8, membrane-associated RING-CH 8; N, nucleocapsid protein; NDP52, nuclear dot protein 52 kDa; PEDV, porcine epidemic diarrhea virus.

PEDV causes diarrhea and vomiting, and death of 50–100% of infected piglets, thus representing a threat to swine industry worldwide. In this study, we investigated the potential host factor modification upon PEDV infection and its regulation during viral replication. We identified RALY as a molecule able to suppress PEDV replication by degrading the viral N protein through the RALY–MARCH8–NDP52–autophagosome pathway (Fig. 5).

As a member of the heterogeneous nuclear ribonucleoprotein family, RALY protein was initially identified as a cross-reacting autoantigen, which could interact with the Epstein–Barr nuclear antigen 1 (EBNA1), a viral protein encoded by the Epstein–Barr virus (25). RALY contains an RNA-recognition motif at the N terminus, two predicted nuclear localization signals, and a glycine-rich region at the C terminus (4), which has been reported to regulate the stability of specific transcripts (11) and participate in transcriptional and post-transcriptional regulation (26). Recent studies reported the association of RALY with CRC stages and aggressiveness, making it a potential therapeutic target for CRC (22). Here, we revealed that PEDV infection inhibited endogenous RALY expression in host cells, and overexpression of RALY suppressed PEDV replication at various time points or infection stages in Vero and LLC-PK1 cells. Furthermore, we found that RALY coimmunoprecipitated with the N protein, and RALY in the nucleus shuttled to the cytoplasm where it colocalized with the N protein. These results indicate that RALY may be a potential therapeutic target for PEDV infection.

Autophagy is a conserved cellular process involving the formation of autophagosomes, which enclose and deliver cytoplasmic cargo (including long-lived proteins, protein aggregates, and organelles) to lysosomes for degradation. Under stress conditions, such as starvation or pathogen infection, host autophagy activity is upregulated, and autophagic cargo is degraded in autophagosomes after fusion with lysosomes (27, 28). We previously reported that the PEDV life cycle is closely related to autophagy (18, 20, 24). In this study, we demonstrated that the degradation of N protein induced by RALY was reversed by autophagy inhibitors BafA1, 3-MA, and CQ. According to the previously reported role of autophagy in resisting PEDV infection (16), we showed that RALY displayed a protective function against PEDV infection by promoting the degradation of viral proteins into the autophagosomes upon PEDV infection.

In a recent report, we have shown that, during infection, host factors can recruit E3 ubiquitin ligase MARCH8 to catalyze the ubiquitination of PEDV N protein, thus inducing selective autophagic degradation (24). MARCH8 is a member of the MARCH E3 ubiquitin ligase family and was reported to inhibit HIV-1 and vesicular stomatitis virus G pseudovirus infection (29, 30). Moreover, Tokunaga *et al*. (31) reported that MARCH8 facilitated the ubiquitination-dependent downregulation of transmembrane proteins. MARCH8 ubiquitinates the PEDV N protein, and the ubiquitinated PEDV N protein is recognized by NDP52, resulting in NDP52-dependent degradation of the N protein through the LC3-mediated autophagy pathway to suppress viral replication in infected cells (24, 31, 32). Here, we found that ablation of either MARCH8 or NDP52 can inhibit the RALY-induced degradation of the PEDV N protein. Furthermore, RALY–MARCH8–NDP52–autophagosome pathway plays a critical role in N protein degradation and protects the host against PEDV infection.

In summary, we have shown that RALY can block PEDV replication by degrading the viral N protein. Upon PEDV infection, RALY recruits the E3 ubiquitin ligase MARCH8 to mediate the ubiquitination of the PEDV N protein; then, the cargo receptor NDP52 recognizes the ubiquitin complex (PEDV N–RALY–MARCH8) and delivers N protein to autolysosomes for degradation. Here, we provide evidence for a novel mechanism of RALY-mediated viral infection control. The role of RALY in protecting the host from PEDV infection may provide the basis for a new potential therapeutic strategy.

## Experimental procedures

### Cell culture, virus, and reagents

HEK 293T cells (American Type Culture Collection; catalog no.: CRL-11268) were cultured in Dulbecco’s modified Eagle’s medium (Sigma–Aldrich; catalog no.: D6429) supplemented with 10% fetal bovine serum (Gibco; catalog no.: 10099141). Vero cells (African green monkey kidney cells; ATCC; catalog no.: CCL-81) were cultured in Dulbecco’s modified Eagle’s medium (Invitrogen; catalog no.: 12430054) with 10% fetal bovine serum. LLC-PK1 cells (porcine kidney cells) were provided by Dr Rui Luo (Huazhong Agricultural University) and maintained in modified Eagle’s medium (Invitrogen; catalog no.: 11095080). All cells were incubated at 37 °C with 5% CO_2_. PEDV strain JS-2013 was isolated and stored in our laboratory.

Anti-RALY antibody (ab170105) was purchased from Abcam. Anti-ACTB/β-actin (66009-1-lg) and anti-GST-tag (10000-0-AP) antibodies were purchased from Proteintech. Anti-HA-tag (3724) and anti-FLAG-tag (F1804) antibodies were purchased from Sigma. Mouse anti-MYC-tag antibodies (2276S), rabbit anti-MYC-tag antibodies (2278S), and BafA1 (54645) were purchased from Cell Signaling Technology. Human NDP52 siRNA (sc-93738) and MARCH8 siRNA (SC-90432) were obtained from Santa Cruz Biotechnology. 3-MA (M9281), chloroquine phosphate (CQ; PHR1258), and MG132 (M7449) were purchased from Sigma–Aldrich. 4′,6-Diamidino-2-phenylindole (C1002) was purchased from Beyotime Biotechnology. The Dual-Glo Luciferase Assay System (DL101) and ClonExpress II One Step Cloning Kit (C112-02) were purchased from Vazyme Biotech Co Ltd. The monoclonal antibody directed against the PEDV (JS-2013) N protein was prepared in our laboratory.

### Transfection and viral infection

Cells were plated in 6-well culture plates and, when at approximately 80–90% confluence, were transfected with the indicated plasmids using Lipofectamine 3000 reagent (Invitrogen; catalog no.: L3000015) according to the manufacturer’s instructions. Cells at approximately 50–60% confluence were transfected with siRNA using Lipofectamine RNAiMAX (Invitrogen; catalog no.: 13778150).

Cells were infected with PEDV at an MOI of either 0.01 or 1 at 37 °C for 1 h. Unattached viruses were removed by washing with PBS solution. The infected cells were then incubated in fresh medium for different periods. The PEDV titer was determined using the median 50% tissue culture infective dose.

### Western blotting

Cells were washed with cold PBS and lysed in radioimmunoprecipitation lysis buffer (Thermo Fisher Scientific; catalog no.: 89901) containing a protease inhibitor cocktail (Bimake; catalog no.: B14001) and phosphatase inhibitor cocktail (Bimake; catalog no.: B15001). Lysates were denatured for 10 min in 5× SDS-PAGE sample loading buffer, separated by SDS-PAGE, and transferred to nitrocellulose Western blotting membranes (GE Healthcare; catalog no.: 10600001). Subsequently, the membrane was washed and incubated with PBS containing 0.2% Tween-20 (Sigma–Aldrich; catalog no.: P1379) and 5% nonfat dry milk (BD; catalog no.: 232100), incubated with the primary antibodies at room temperature, followed by horseradish peroxidase–conjugated secondary antibodies (Proteintech Group; catalog no.: SA00001-1), and detected with enhanced chemiluminescence (Share-bio; catalog no.: SB-WB012).

### qRT–PCR

Total RNA was isolated using the QIAamp Viral RNA Mini Kit (Qiagen; catalog no.: 52906) or RNeasy Mini Kit (Qiagen; catalog no.: 74104), according to the manufacturer's instructions and reverse transcribed to generate complementary DNA using the PrimeScript RT reagent Kit (Takara; catalog no.: RRO47A). qRT–PCR was performed using SYBR Premix Ex Taq (Vazyme Biotech Co, Ltd; catalog no.: q711-03). All data were normalized to the β-actin reference gene. The primers used are listed in Table S1.

### co-IP assay

Transfected cells were incubated for 24 h and then lysed in NP40 cell lysis buffer (Life Technologies; catalog no.: FNN0021) containing a Protease Inhibitor Cocktail, followed by incubation with anti-FLAG-antibody-bound Dynabeads Protein G (Life Technologies; catalog no.: 10004D). Subsequently, the supernatants were washed thrice with 0.02% PBS with Tween-20 and resuspended in elution buffer (50 mM glycine, pH 2.8). Proteins were analyzed by immunoblotting with the indicated antibodies.

### GST affinity–isolation assay

PEDV *N*, *MARCH8*, *NDP52*, and *RALY* gene sequences were cloned into either pCold GST plasmid (Clontech Laboratories, Inc; catalog no.: 3372) or pCold TF plasmid (Clontech Laboratories, Inc; catalog no.: 3365) and expressed in BL21-competent cells (Vazyme Biotech; catalog no.: C504-03). The GST Protein Interaction Pull-Down Kit (Thermo; catalog no.: 21516) was used to analyze protein interactions, according to the manufacturer’s instructions. The proteins were eluted with reduced glutathione and analyzed by Western blotting.

### Confocal immunofluorescence assay

HeLa cells were transfected for 24 h, washed, fixed with 4% paraformaldehyde (Sigma–Aldrich; catalog no.: P6148), and permeabilized with 0.1% Triton X-100 (Sigma–Aldrich; catalog no.: T9284) for 1 h at room temperature. Subsequently, cells were blocked with 5% bovine serum albumin (Cell Signaling Technology; catalog no.: 9998) for 1 h and incubated with the primary antibody for 1 h. After three washes with PBS, cells were incubated with a fluorescence-labeled secondary antibody for 1 h in the dark, as previously described (24). Nuclei were stained with 4′,6-diamidino-2-phenylindole (catalog no.: C1002) for 5 min. Fluorescence images were captured using a laser-scanning confocal immunofluorescence microscope (Carl Zeiss).

### Statistical analysis

Group comparisons were conducted using two-tailed Student’s *t* test with GraphPad Prism 5 software (GraphPad Software, Inc). ∗*p* < 0.05, ∗∗*p* < 0.01, and ∗∗∗*p* < 0.001 were considered statistically significant; ns stands for not significant. All results are representative of three independent experiments.

## Data availability

All data are contained within the article.

## Supporting information

This article contains supporting information.

## Conflict of interest

The authors declare that they have no conflicts of interest with the contents of this article.
