# Supplementary material for: Nuclear ribonucleoprotein RALY targets virus nucleocapsid protein and induces autophagy to restrict porcine epidemic diarrhea virus replication
Source: J Biol Chem. 2022 Jun 24;298(8):102190. doi: 10.1016/j.jbc.2022.102190 (PMC9287142; doi:10.1016/j.jbc.2022.102190)
Supplement: Table S1 [file mmc1.docx]

**Table S1.** List of primer and siRNA sequences used in this study.

| Purpose | names | Sequence (5′-3′) |
| --- | --- | --- |
|  | PEDV *N* forward | GAGGGTGTTTTCTGGGTTG |
|  | PEDV *N* reverse | CGTGAAGTAGGAGGTGTGTTAG |
|  | *pRALY* forward | AGAGCCCAAGCCCAACAGA |
| Real-time PCR | *pRALY* reverse | TCGTAGAAGTCGTCCCGGTAG |
| Primers | *ACTB* forward | TCCCTGGAGAAGAGCTACGA |
|  | *ACTB* reverse | AGCACTGTGTTGGCGTACAG |
|  | *pGAPDH* forward | ATGGATGACGATATTGCTGCGCTC |
|  | *pGAPDH* reverse | TTCTCACGGTTGGCTTTGG |
|  | *si-RALY* sense | CCACCAUACCUGUCAAGCUTT |
|  | *si-RALY* antisense | AGCUUGACAGGUAUGGUGGTT |
| siRNA sequences | NC sense | UUCUCCGAACGUGUCACGUTT |
|  | NC antisense | ACGUGACACGUUCGGAGAATT |
